# The resistome and genomic reconnaissance in the age of malaria elimination

**DOI:** 10.1242/dmm.040717

**Published:** 2019-12-19

**Authors:** Krittikorn Kümpornsin, Theerarat Kochakarn, Thanat Chookajorn

**Affiliations:** 1 Parasites and Microbes Programme, Wellcome Sanger Institute, Wellcome Genome Campus, Hinxton CB10 1SA, UK; 2 Genomics and Evolutionary Medicine Unit (GEM), Centre of Excellence in Malaria Research, Faculty of Tropical Medicine, Mahidol University, Bangkok 10400, Thailand

**Keywords:** Drug resistance, Genomic epidemiology, Malaria

## Abstract

Malaria is an infectious disease caused by parasitic protozoa in the *Plasmodium* genus. A complete understanding of the biology of these parasites is challenging in view of their need to switch between the vertebrate and insect hosts. The parasites are also capable of becoming highly motile and of remaining dormant for decades, depending on the stage of their life cycle. Malaria elimination efforts have been implemented in several endemic countries, but the parasites have proven to be resilient. One of the major obstacles for malaria elimination is the development of antimalarial drug resistance. Ineffective treatment regimens will fail to remove the circulating parasites and to prevent the local transmission of the disease. Genomic epidemiology of malaria parasites has become a powerful tool to track emerging drug-resistant parasite populations almost in real time. Population-scale genomic data are instrumental in tracking the hidden pockets of *Plasmodium* in nationwide elimination efforts. However, genomic surveillance data can be useful in determining the threat only when combined with a thorough understanding of the malarial resistome – the genetic repertoires responsible for causing and potentiating drug resistance evolution. Even though long-term selection has been a standard method for drug target identification in laboratories, its implementation in large-scale exploration of the druggable space in *Plasmodium falciparum*, along with genome-editing technologies, have enabled mapping of the genetic repertoires that drive drug resistance. This Review presents examples of practical use and describes the latest technology to show the power of real-time genomic epidemiology in achieving malaria elimination.

## Abstract (precis)

**Summary:** This Review discusses the challenges in malaria elimination and how implementation of national-scale genomic surveillance programmes in combination with resistome analyses could provide a powerful solution.

## Introduction

Malaria is a serious mosquito-borne tropical infectious disease caused by protozoa in the *Plasmodium* genus. Malaria elimination (see Glossary, Box 1) has become a real possibility once again after previous attempts have proven that elimination of malaria parasites on a global scale is elusive (Li et al., 2019). The new hope springs from an unprecedented scale of investment and commitment, resulting in new drug candidates and genetic toolkits considered unattainable just a decade ago. A new paradigm of nearly real-time genomic epidemiology has provided insights into emerging populations of drug-resistant parasites almost at the same time as their impact on the treatment outcome was recognized. The availability of large-scale genomic data arising from extensive surveillance networks is a critical element in the development of an effective reconnaissance strategy to ‘search and destroy’ every pocket of parasite transmission. Failure to respond to the looming threat from malaria drug resistance with effective treatment regimens and control measures would permit residual parasites to evade the elimination efforts. Thus, elimination plans can only succeed when the genomic surveillance data are paired with information on the molecular mechanisms underlying the modes of action, and the resistance mechanisms associated with existing antimalarial drugs and the new candidates in the research and development (R&D) pipeline.

**Box 1. Glossary**

**Antifolate**: the drug family that inhibits folate production, a key substrate in DNA synthesis, making the drugs effective against actively propagating targets such as bacteria, cancer cells and parasites. The antimalarials pyrimethamine and sulfadoxine are antifolates.

**Artemisinin**: a sesquiterpene lactone compound with a peroxide bridge, originally extracted from *Artemisia annua*. It is a fast-acting antimalarial drug currently used for treating malaria worldwide. The success of this drug is one of the reasons behind the recent reduction in malaria mortality.

**Chloroquine**: a 4-aminoquinoline antimalarial drug that used to be a part of the treatment regimens due to its fast-acting and safety profile. Currently, the drug is ineffective for treating drug-resistant *P. falciparum* malaria, but is still the drug of choice for *P. vivax* malaria.

**Druggable space**: a compendium of biomolecules, mostly proteins that can be perturbed by (small) molecules to achieve desirable pharmacological properties. Not every essential gene product is druggable, since putative targets must provide a certain degree of specificity to avoid cross-reaction and toxicity.

**Kelch**: a large protein family containing the Kelch motif, a blade-like structure built from beta-sheets to form a propeller. A member of this family, Kelch13, has been linked to artemisinin resistance.

**Malaria elimination**: the reduction of locally transmitted malaria cases to zero in a defined geographical region. Any imported case needs to be managed quickly. This term should not be confused with eradication, which is a worldwide goal of zero incidence of malaria cases. Eradication can be achieved when the regional elimination efforts have been achieved.

***piggyBac***: a mobile transposable element originally isolated from a moth species. In conjunction with a transposase, it is a useful tool in genetic engineering due to its strong insertional mutagenesis activity.

**Resistome**: the compendium of all resistance genes including the precursor genes capable of evolving to cause drug resistance.

**Tajima's D**: a statistical test named after Fumio Tajima. It determines and compares the degree of genetic diversity in the population scale. The test can be used to indicate an evolutionary process under selection.

**Saturation screen**: a screen for the genetic factors capable of functionally perturbing a phenotype of interest. The screen is supposed to reach saturation when all genes are experimentally tested.

**Zinc finger**: one of the DNA-binding protein motifs. Its zinc-coordinated scaffold interacts with DNA with high specificity, which could be rationally engineered to recognize a specific region. It is the key sequence-recognition subunit of zinc finger nucleases (ZFNs), which can be customized to cut DNA at a predetermined site, enabling precise genome editing.

In recent years, novel genetic toolkits in combination with the classic long-term drug selection approach and the analysis of drug resistance evolution have become powerful tools in identifying drug targets. These approaches have also shed light on the evolutionary process by which parasites overcome antimalarial drugs. A series of long-term selection experiments have proven to be useful beyond drug target identification. On the surface, drug target identification and genomic surveillance are not related, but the two fields have become intertwined, affecting global malaria control efforts. Genome-wide screens exploring potential drug targets and resistance mechanisms have also extensively investigated their putative evolutionary paths to the point at which the data can now be combined with genomic surveillance to identify emerging parasite populations that carry existential threats to malaria treatment and control. This Review discusses how these powerful molecular toolkits can work side by side with genomic surveillance to accomplish malaria elimination.

## A brief history of antimalarial drugs and their resistance

Malaria has left its footprint throughout the history of human civilization. Even during the past few hundred years, eight Presidents of the United States, including George Washington, were afflicted by the disease (https://www.impatientoptimists.org/Posts/2012/01/Cherry-Trees-Log-Cabins-and-Malaria). At present, almost half of the world population still live under the threat of malaria, with over 200 million cases every year, and antimalarial drugs remain the only proven effective means to treat patients (https://www.who.int/malaria/publications/world-malaria-report-2018/en/). The medical and research communities are currently working towards malaria elimination, and have already achieved a promising global reduction in the malaria mortality rate. However, almost all effective antimalarial drugs have been lost to drug-resistant parasites (White et al., 2014). Even though more new families of antimalarial drug candidates are available in the pipeline now than ever before, the emergence of new multidrug resistance in parasites could negate years of elimination efforts (Hooft van Huijsduijnen and Wells, 2018).

**Fig. 1.**
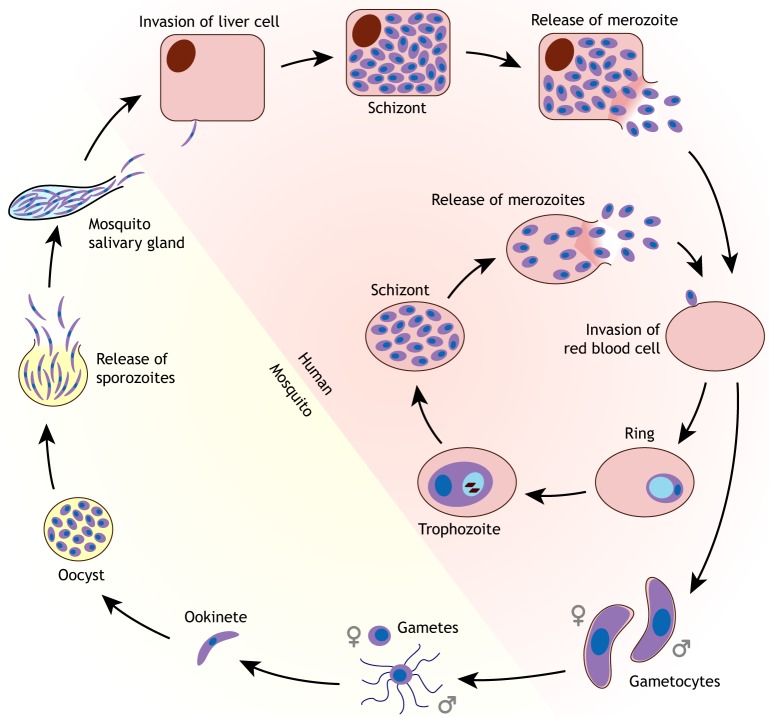
**Malaria life cycle.** The parasitic protozoa switch between the vertebrate and the mosquito hosts. The sporozoite released via a mosquito bite enters the liver cell of the vertebrate host to produce progenies for invading the red blood cells. The majority of the parasites undergo the asexual cycle inside the vertebrate host. A portion of the parasites enter the sexual cycle, producing gametocytes for mating inside the mosquitoes. Most antimalarial drugs kill the blood-stage asexual parasites. Only primaquine and its derivatives (Box 1) are capable of killing the liver-stage and gametocytic stages of the parasites, thus preventing further transmission to the mosquito host.

Human malaria is caused by the single-celled parasites in the genus *Plasmodium*; namely, *Plasmodium falciparum*, *Plasmodium vivax*, *Plasmodium malariae* and *Plasmodium ovale* (White et al., 2014). The zoonotic *Plasmodium knowlesi*, a monkey malaria parasite, was found to be able to infect humans living near the forest in Southeast Asia (Cox-Singh et al., 2008). Malaria parasites are transmitted between humans via blood feeding of female mosquitoes in the *Anopheles* species complex (Fig. 1) (Phillips et al., 2017). After entering the human host, the parasites first invade liver cells, multiply and egress to infect red blood cells (Fig. 1) (Phillips et al., 2017). The parasitic protozoa propagating and residing inside the human red blood cells are responsible for most of the malaria-related symptoms. *P. falciparum* malaria is the deadliest form of human malaria because patients can develop severe symptoms including coma, organ failure and anaemia (White et al., 2014).

During the 1960s, the availability of chloroquine and antifolates (Box 1), in addition to active dichlorodiphenyltrichloroethane (DDT) mosquito spraying, gave an illusion of worldwide malaria elimination (Nájera et al., 2011). However, the failure of this elimination campaign resulting from the spread of multidrug-resistant parasites was a rude awakening, demonstrating how a few mutations in the parasite population can turn the tide, with millions of human lives lost as a result (White, 2012). Half a century later, our generation is equipped with several new drugs, giving us the same opportunity to eliminate malaria and to inevitably face the same challenge from new drug-resistant *Plasmodium* strains. During the past 50 years, studies on malaria drug resistance have focused on chloroquine, antifolate and, more recently, artemisinin (Box 1). Chloroquine and antifolate saved millions of lives during the primes of their clinical uses. At present, artemisinin combination therapy is considered the most effective regimen for *P. falciparum* malaria treatment (White, 2008). The drug was found to be highly effective in clearing the parasites, but has a short half-life. It therefore requires co-administration with another antimalarial drug with a long half-life. The key to the success of artemisinin is its fast parasite clearance time. Its implementation has saved millions and has become a hallmark for the triumph of traditional medicine in modern time (Su and Miller, 2015). The first sign of artemisinin resistance has recently emerged in malaria cases that require more time to clear the parasites when exposed to artemisinin. The delayed clearance following artemisinin treatment was first observed in few cases at the border between Cambodia and Thailand, but these parasites have now spread to many Greater Mekong Subregion countries in Southeast Asia (Amato et al., 2018; Dondorp et al., 2009; Imwong et al., 2017). It is obvious that the problem is growing, but the current artemisinin regimen and dosage can still treat malaria patients (Chookajorn, 2018). However, the spread of full-blown resistance to Africa could cost millions of lives. To prevent this catastrophe, drug efficacy and genomic surveillance projects have been ongoing in Southeast Asia. Control efforts also include the implementation of new and additional partner drugs to artemisinin. Mefloquine, piperaquine and lumefantrine have been the partner drugs of choice in Cambodia, Thailand and Laos, respectively. Pyronaridine is also being explored in a limited area. These partner antimalarial drugs are vital in protecting artemisinin from drug resistance, and any sign of ineffectiveness must be detected as quickly as possible. Nevertheless, little information is available for tracking drug targets and resistance mechanisms, and this includes drug candidates in the antimalarial R&D pipeline.

## Exploring the resistome for malaria elimination

**Fig. 2.**
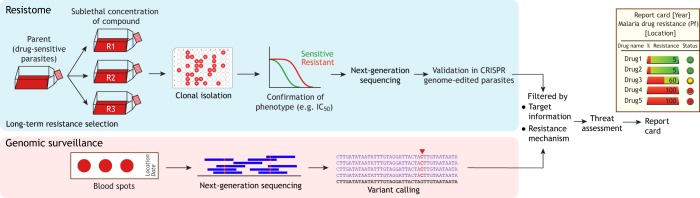
**Combination of long-term selection and genomic surveillance workflows.** The selection workflow in the light-blue box represents a series of selections performed in parallel (R1, R2 and R3). The surviving parasites are cloned and tested for changes in drug sensitivity levels as determined by the half-maximal inhibitory concentration (IC_50_) analysis. The mutations arising from this selection process, especially those found in all the parallel selection experiments, are functionally validated by genome editing. The list of mutations can be used as a filter to flag emerging variants from genomic surveillance efforts in the field that use blood spot samples from malaria patients. These variants will be closely monitored and experimentally tested for their ability to alter drug sensitivity levels. This surveillance data will be converted into a report card summarizing the drug sensitivity profile and the parasite population structure to inform malaria treatment and elimination efforts.

Long-term resistance selection is the experimental method of choice for target identification in malaria research (Luth et al., 2018). The selection process also reveals the resistance mechanisms for different antimalarial drugs. This drug pressure selection approach was originally employed with rodent and avian malaria parasites (Rollo, 1952), where drug-sensitive isolates were exposed to antimalarial drugs to select for resistant clones. For example, selection with antifolate pyrimethamine in *Plasmodium chabaudi* and *Plasmodium yoelii* gave rise to a mutation in the gene encoding dihydrofolate reductase, the target of pyrimethamine, and successfully selected for the S106N mutation, the equivalent of the critical pyrimethamine-resistance-associated S108N mutation in *P. falciparum* (Cheng and Saul, 1994; Cowman and Lew, 1990; Peterson et al., 1990). The modern standard for long-term drug selection relies on an *in vitro* culture system. The first *in vitro* resistance selection experiment in *P. falciparum* was reported in 1978 by continuously exposing the African FCR-3 line to chloroquine, yielding a stable phenotype capable of tolerating higher doses of chloroquine (Nguyen-Dinh and Trager, 1978). This finding forewarned the fate of chloroquine treatment in Africa. The common selection method is performed in batches and exposing the parasites to the drug either continuously or in pulses (Fig. 2). The surviving parasites are supposedly selected by acquiring mutations in the genes encoding either the drug target or a resistance mechanism. The individual resistant clones are isolated to ensure genetic homogeneity and validated by dose-response analysis. This approach reliably elucidated and confirmed the drug targets and the resistance pathways of multiple novel drug candidates in the pipeline (Table 1; the rationale behind target identification is explained in Box 2). From the examples shown in Table 1, there are several factors that could shape the selection process, including the drug concentration (incremental or fixed) and the exposure time (pulsed or prolonged) (Nzila and Mwai, 2010). There is no definite rule on the best selection protocol, and the selection workflow must be optimized for each drug.

**Table 1.**
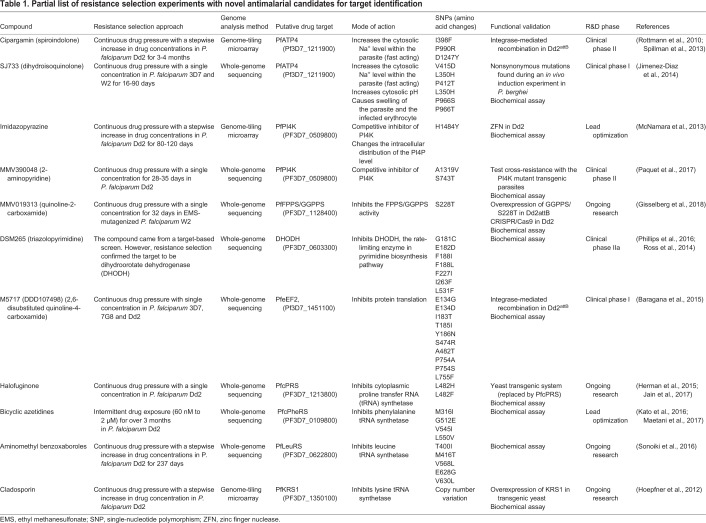
**Partial list of resistance selection experiments with novel antimalarial candidates for target identification**

**Box 2. Why we still need new drugs and more targets for malaria elimination**

Malaria is eliminated when there are no new cases from local transmission. During the past half-century, several countries, especially in the developed world, successfully eliminated malaria (Li et al., 2019). However, the elimination efforts at the epicentre of multidrug-resistant malaria in Southeast Asia have not been successful yet (Newby et al., 2015; Smith Gueye et al., 2014). A major limitation in malaria elimination is the failure to systemically kill parasites in two *Plasmodium* developmental stages; namely, the sexual gametocyte and liver stages (Fig. 1). This is one of the reasons why the modern-day standard for an ideal antimalarial drug requires multi-stage killing capacity with the emphasis on liver hypnozoites and sexual gametocytes (Burrows et al., 2017). Activities against both stages are instrumental in the global malaria elimination effort because the parasites in the liver hypnozoite form can lay hidden for years, and the unchallenged transition to sexually committed gametocytes could become an escape route to reach the mosquito host for another round of mating. The success of malaria elimination in developed countries relies on effective control measures blocking the cycle of transmission and preventing the outbreak from imported cases, a luxury unavailable to countries with limited resources and porous borders. The multiple candidate drug families currently under development are promising. As the targets for many of these drug candidates were already identified, one might ask why target identification is still needed. The answer to this question lies in the fact that many attractive drug candidates were discovered through high-throughput screening (Gamo et al., 2010; Guiguemde et al., 2010). The chemical libraries used for screening remain largely untapped resources that contain the compounds with desirable drug properties, and knowing their potential targets will allow researchers to monitor for drug resistance evolution. Another benefit of target identification is the opportunity to explore the druggable space of the precious known antimalarial drugs. For example, primaquine is the only drug capable of killing the liver-stage and gametocytic stages (Graves et al., 2018). Its 8-aminoquinoline scaffold is similar to a more classical antimalarial 4-aminoquinoline found in the widely used chloroquine, piperaquine and mefloquine (Vale et al., 2009). The mystery is why a simple switch in the position of a nitrogen atom in the quinoline scaffold could broaden its antimalarial activity. This question has a larger implication beyond an academic exercise. Even though primaquine is an ideal drug for radical treatment, it can cause a severe adverse effect in individuals with glucose-6-phosphate dehydrogenase (G6PD) deficiency (Howes et al., 2013), where the presence of metabolized primaquine was proposed to instigate oxidative damage, especially to the red blood cells with a compromised redox system from low G6PD (Ganesan et al., 2012). Conventional structure and activity relationship (SAR) analysis is a strategy for identifying the core pharmacophore conferring the drug activity and for removing undesirable chemical moieties causing adverse effects. Implementing this approach for primaquine would allow the development of its pharmacophore scaffold toward a safer drug for the population with G6PD deficiency. The question is how one can mimic the activity of 8-aminoquinoline. Unlocking the secret behind its unique killing activity requires the understanding of the mode of action and its putative targets.

The mutations found after drug selection need to be functionally validated. The malaria research field was limited by the availability of molecular tools to specifically modify the candidate genes. The application of zinc finger (Box 1) nuclease (ZFN) in *P. falciparum* provided a possible solution (Straimer et al., 2012). However, each ZFN needs to be custom tailored to the target DNA base pairs, making cost and reliability the major issues limiting widespread adoption (Bibikova et al., 2003; Shaw and Aroonsri, 2017). Recently, testing the functional significance of each variant has become easier and faster by the application of clustered regularly interspaced short palindromic repeats (CRISPR)/Cas9 genome editing in *P. falciparum*. This system can achieve genome editing in *P. falciparum* by introducing a point mutation and disrupting the gene of interest (Ghorbal et al., 2014; Wagner et al., 2014). The method requires three major components; namely, the *Streptococcus pyogenes* Cas9 (*Sp*Cas9) endonuclease, a single-guide RNA (sgRNA) and a donor DNA template. The sgRNA is designed to lead *Sp*Cas9 to the target genomic site using complementary base pairing. The donor template containing a mutation of choice is introduced into the genome at the *Sp*Cas9-induced breakage site by homology-directed repair. The benefit of CRISPR/Cas9 compared to conventional recombinant DNA technology is that the former does not leave an extra plasmid component in the chromosome. The dependency on matching nucleotides provides a cost-effective and versatile system for gene editing, surpassing ZFN. The feasibility of CRISPR/Cas9 in *P.* *falciparum* was first proven by testing the effect of *kelch13* mutations (Box 1) on parasite susceptibility to artemisinin (Ghorbal et al., 2014). Subsequently, the CRISPR/Cas9 machinery was proven to be functional in *P. falciparum* without plasmid transformation: Crawford et al. (2017) used the Cas9-sgRNA ribonucleoprotein complex with a single-stranded oligodeoxynucleotide as a donor template to introduce mutations in the gene encoding the PfATP4 sodium transporter. As PfATP4 is the target of the new fast-acting antimalarial drug candidate SJ733, the mutations introduced by this plasmid-free approach reduced the antimalarial effect of SJ733 as expected. In addition, applying selection-linked integration (SLI) can significantly speed up the selection process of edited parasites (Birnbaum et al., 2017). SLI is a plasmid-based system that relies on the integration-linked resistance marker located at the 3′ end of the homologous template with a self-cleavage 2A peptide to separate the modified protein and the drug-resistant selection protein. This technique can improve the rate of the gene-editing process from months to just a few weeks, rendering the discovery of putative genetic markers of resistance more efficient.

Interestingly, the ease of genome editing in *P. falciparum* has expanded the scope of druggable space (Box 1) and resistance mechanisms. A network of researchers have systematically chosen potent antimalarial compounds and performed drug selection on a large scale (Antonova-Koch et al., 2018; Corey et al., 2016; Cowell et al., 2018). The emerging mutations were tested for their ability to shift drug sensitivity. The studies revealed new putative targets, including aminophospholipid-transporting P-type ATPase and farnesyltransferase (Cowell et al., 2018), and the approach has now been expanded to target multiple *Plasmodium* life-cycle stages, especially the sexual and liver stages, with a larger drug candidate collection (Antonova-Koch et al., 2018). A companion approach is genome-wide mutation screening, which has also played a prominent role in mapping drug targets and resistance mechanisms during the past 2 years. The *piggyBac* (Box 1) system uses a transposable element to disrupt functional genes (Balu et al., 2005). A saturation screen (Box 1) was performed by allowing *piggyBac* elements to randomly insert into the malarial genes and determine their dispensability. A collection of parasite clones with *piggyBac* insertion at various genes (∼40% of the *P. falciparum* genes) was exposed to artemisinin, and the abrogation of the genes related to the drug's mode of action and cellular response affected the drug sensitivity level (Zhang et al., 2018). This gene-based approach successfully identified the role of the proteasome degradation pathway, including that of Kelch13, in artemisinin sensitivity (Zhang et al., 2018). A similar large-scale knockout screen in *Plasmodium berghei*, a murine malaria parasite known to be more genetically malleable than its human counterparts, revealed the degree of dispensability of almost every malarial gene during the asexual blood stage (Bushell et al., 2017). These recent large-scale projects have allowed saturation screening to reach levels not possible in malaria research during the past decades and have opened up the druggable space, including the putative resistome of malaria parasites (Cowell and Winzeler, 2018). The concept of resistome (Box 1) originally arose from studies of bacterial antibiotics (Wright, 2007). The size and diversity of genomic data accelerate the discoveries of resistance genes and their precursors (Crofts et al., 2017). Structural and expression changes of the drug target are the most common evolutionary pathways to resistance. However, proto-resistance genes that have no direct role in the biochemical and physiological pathway could evolve to reduce the collateral biological damage caused by the drug (Crofts et al., 2017). Predicting all possible evolutionary pathways towards drug resistance in *Plasmodium* might seem farfetched, but recent reports on both long-term selection and genome-wide screening have already generated a plethora of data. The genes and variants identified in these studies could be developed into a sentinel filter, identifying the potential threats of emerging genetic variants as part of the global genomic malaria surveillance effort. For example, mutations in the genes encoding the ABC transporter PfABCI3 and amino acid transporter PfAAT1 were associated with reduced sensitivity for several antimalarial drug candidates (Cowell et al., 2018). These genes were not previously known to be linked with drug resistance, but their functions and broad biological effects warrant further functional studies.

## Combining resistome research and genomic surveillance in malaria elimination

**Fig. 3.**
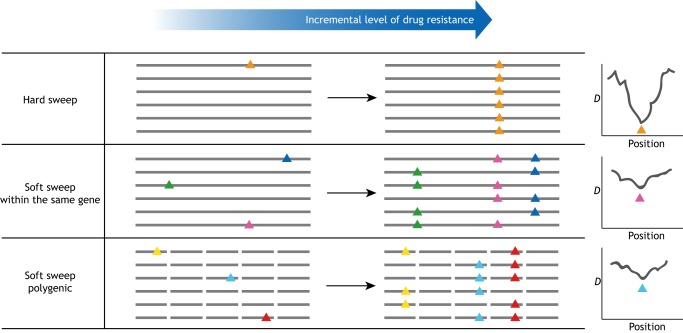
**Hard and soft sweeps in drug resistance evolution.** In the hard sweep scenario, the evolutionary process toward a drug-resistant phenotype can be driven by a few mutations, and they quickly dominate over other alleles in a population, giving a strong selective signature, as shown with a negative Tajima's D value (D). However, the evolutionary process is often less than ideal, and the accumulation of several mutations either within the same gene or in multiple genes contribute to the eventual drug resistance outcome, making the selective signature less obvious and less easy to track.

In order to appreciate the power of the malarial resistome in elimination efforts, the broader community needs to become aware of the unprecedented large-scale genomic surveillance efforts in several endemic countries. These involve mass blood spot collection from patients, making the malaria genome database one of the best genomic databases in terms of diversity and size (Box 3). Despite the initial success, implementation of genomic reconnaissance in malaria elimination needs to be precise and almost in real time to root out the last pockets of parasite populations. It is not an overstatement to say that the ‘last mile’ of malaria elimination is likely to be the hardest. Parties involved in malaria elimination can be lulled into false security if residual parasite populations eluding the efforts linger unnoticed in asymptomatic individuals and re-emerge once control measures are removed. The current phase of malaria genomic surveillance is fostered by collaborations at the national level. The GenRe-Mekong programme works with national malaria control programmes in the Greater Mekong Subregion in Southeast Asia to genotype malaria parasites nationwide in each country. The genomic data are translated into a report card summarizing drug sensitivity profile and population movement (Fig. 2). Actionable epidemiology data are purported to be a guide in malaria control and elimination for the national public health authorities based on the effective treatment regimen for each area and the origin of the circulating parasites. However, the scale of genomic epidemiology surveillance also means that a plethora of genetic variants that naturally emerge in *Plasmodium* populations will be reported. In the past, genetic association studies have identified mutations with strong linkage to drug resistance, revealing the causal mutations and/or putative resistance markers (Wang et al., 2016). The emerging variants are often fixed as dominating alleles due to their selective advantage from either conferring drug resistance or fitness compensation (Chookajorn and Kumpornsin, 2011). Routine genomic epidemiology surveillance will detect emerging variants instantaneously even before they become fixed in the population. In the most straightforward scenario, a specific drug-resistant allele is favoured within a population and undergoes a ‘hard’ sweep into fixation (Fig. 3) (Pritchard et al., 2010). However, several mutations could emerge in response to drug selection, and multiple evolutionary paths towards drug resistance or any advantageous traits could occur simultaneously without a clear winner. More than one single genetic variant from multiple genes or even within the same gene can accumulate over time. The ‘soft’ sweep scenario means that no clear selective signal will be detected during genomic surveillance, and it will take a relatively long time before a particular allele becomes a major feature in a parasite population (Fig. 3) (Pritchard et al., 2010). The common parameters for determining selection, such as Tajima's D (Box 1), are less useful in tracking the soft sweep (Fig. 3) (Messer and Petrov, 2013). The foreseeable challenge for large-scale genomic surveillance projects is to evaluate the rise of emerging variants in the context of antimalarial drug resistance. In fact, real success would be to identify potential threats from emerging alleles and to suppress them before a strong drug-resistance driver becomes fixed in the parasite population.

**Box 3. A genetic variant call and database for the malaria research community**

At the start of malaria genomic research, the identification of *P. falciparum* genetic variants was achieved by polymerase chain reaction (PCR) amplification of the target region followed by Sanger sequencing. This workflow was sufficient for studying known polymorphic sites, but is not suitable for novel variant discovery. The implementation of next-generation sequencing technology has paved the way to track new variants on the genomic scale. The most popular next-generation sequencing technology for *P. falciparum* is the Illumina-based method. This method has satisfactorily produced the read quality suitable for the AT-rich *P. falciparum* genome. For the discovery of structural variants (duplications, large indels and translocations), Illumina sequencing can be combined with either nested PCR coupled to Sanger sequencing or with long-read sequencing methods such as PacBio. The long reads from PacBio can extend across the AT-rich regions, making the assembly of the whole chromosome possible (Otto et al., 2018). For genes known to be associated with drug resistance, an amplicon sequencing approach targets the regions of interest for sequencing, reducing the cost and the error by performing sequencing of indexed samples in a large batch.

Global collaborations have resulted in several *Plasmodium* variant collection databases. PlasmoDB (https://plasmodb.org/) provides access to the variant data from laboratory isolates and parasite collections. The *P. falciparum* Community Project (https://www.malariagen.net/projects/p-falciparum-community-project) and the Pf3k project (https://www.malariagen.net/projects/Pf3k) contain the variant and the geographical data from *P. falciparum* across the globe (Malaria Genomic Epidemiology Network, 2008). The January 2016 release of the *P. falciparum* Community Project contains genotype data from 3394 samples from 22 countries. The Pf3k project is a collaboration of research groups at the Broad Institute, the University of Oxford and the Wellcome Sanger Institute to comprehensively analyse the genome variations. The current release (Pf3k pilot data release 5) covers 2512 field isolates, five clonal samples of laboratory-adapted strains, 96 samples from the progenies of genetic crosses and 27 mixed samples of laboratory strains experimentally mixed together as controls. These databases are continuously updated, and additional data for further analysis can be found on their respective websites.

Functional validation, using the techniques discussed above, of emerging variants is instrumental in making sense of the surveillance data. Predicting the functional significance of emerging variants based on known resistome repertoires could provide a solution to this problem. This notion is not a mere abstract concept. In the early days of emerging artemisinin resistance, there was no clear genetic variant to be used as a candidate artemisinin resistance marker. It was obvious at the time that the degree of diversification suggested a strong selective pressure on the parasite populations in Cambodia, the site of emerging artemisinin resistance (Miotto et al., 2013). Genome-wide association studies identified candidates and narrowed the linkage sites to certain chromosomes (Cheeseman et al., 2012). An important consideration for this discussion is that emerging artemisinin resistance does not fit a conventional drug resistance phenotype (Chookajorn, 2018), which is defined as treatment failure at the recommended dosage and treatment regimen. Artemisinin and its derivatives can still kill parasites at the recommended dose, but the clearance time is delayed by hours (Ashley et al., 2014). The thorough and well-organized drug efficacy monitoring effort in Cambodia revealed a clear shift in clearance time, but the problem has been detected before the parasites reached full drug resistance (Ashley et al., 2014). As this ongoing artemisinin resistance evolution did not yet reach the end stage, the selection for a resistance genetic marker was less obvious than in typical full-blown drug resistance. The populations of *P. falciparum* in Cambodia originally emerged as multiple sympatric subpopulations (Miotto et al., 2013). They later evolved towards a shared genetic background with many genetic factors predisposing for artemisinin resistance (Miotto et al., 2015). To provide functional clues, researchers conducted a long-term selection experiment. A *P. falciparum* isolate from Africa was subjected to intermittent artemisinin pressure for 5 years (Ariey et al., 2014). During the selection process, parasites were periodically sampled for artemisinin sensitivity testing and genomic sequencing. The accumulation of novel mutations causing the gradual loss of artemisinin susceptibility revealed a series of evolutionary changes needed for emerging artemisinin resistance. As expected, multiple mutations arose throughout the selection period. The list of genes and mutations by itself was not informative, but, after matching them to the variants found in Cambodia, *kelch13* mutations have become a notable candidate. By the degree of association alone, variants in other genes in the Cambodian population had statistical significance, but they were not found in the selection experiment. CRISPR/Cas9 genome editing experiments confirmed the effect of *kelch13* mutations on improving parasite survival under artemisinin pulses (Ghorbal et al., 2014). *kelch13* mutations alone are not likely to be the sole driver of artemisinin resistance, but the implementation of *kelch13* mutations as a molecular marker played a pivotal role in tracking the spread of the parasites with reduced artemisinin sensitivity (Amato et al., 2018; Imwong et al., 2017; Sa et al., 2018; Wilairat et al., 2016). It is not yet possible to ignore the foreseeable threat of full-blown artemisinin resistance (Chookajorn, 2018). Nevertheless, the studies on *kelch13* and its role in emerging artemisinin resistance have undoubtedly established a new paradigm for a combination of genomic surveillance in the field, with long-term drug selection and genome-editing experiments to prevent and mitigate the spread of drug-resistant pathogens.

The resistome data described above could be used to construct a filter to assess the emerging variants in different parasite populations. Mutations in the genes considered to be components of the malarial resistome need to be subsequently functionally validated, perhaps by CRISPR technology. One possible challenge is to validate a specific mutation among various genetic backgrounds. If parasites with a similar genetic background are cultivated, it will be ideal to introduce mutations in the same background to exclude any confounding effect from genetic interactions. Nevertheless, emerging variants might not be causal, and their roles in fitness compensation must be carefully considered. The story of *kelch13* mutations provides an excellent example. These mutations alone have a relatively weak drug resistance selection effect, but they fit the clinical phenotype of artemisinin resistance. At this point, the significance of *kelch13* as a molecular marker has already contributed significantly to the utility of the resistome in genomic surveillance and malaria elimination.

## The ‘last mile’ of malaria elimination

It is undeniable that good science is vital to malaria elimination, but science alone will not translate into successful elimination. The challenge is to share the scientific data with national and regional control programmes, and to present the shared data in an actionable format beyond gigantic DNA sequence files and variant collections (Dalmat et al., 2019). Translation and distribution of big data in an actionable form such as a genetic report card would ensure that the genomic surveillance data become a part of the decision-making process in a national malaria elimination policy. It also means that the national control programmes in each malaria-endemic country have to form a local team capable of understanding the basic concepts of genomic epidemiology and implement it in an existing public health structure. This requires a capacity-building scheme to train the local workforce, either within a degree-granting system or as short-course training. The structure for any genomic surveillance programme is sustainable only when local workforce feels empowered and appreciates how the genetic data gathered benefit the health and welfare of their fellow compatriots.
